# Shallow population structure of southern bluefin tuna, *Thunnus maccoyii* (Castelnau, 1872) between Indian and Atlantic Oceans inferred from mitochondrial DNA sequences

**DOI:** 10.1080/23802359.2021.1959455

**Published:** 2021-08-02

**Authors:** Jeong Eun Ku, Jin-Koo Kim, Doo Nam Kim, Sung Il Lee

**Affiliations:** a Distant Water Fisheries Resources Research Division, National Institute of Fisheries Science, Busan, Korea; b Department of Marine Biology, Pukyong National University, Busan, Korea

**Keywords:** Southern bluefin tuna, *Thunnus maccoyii*, genetic diversity, population structure, mitochondrial DNA

## Abstract

Southern bluefin tuna (*Thunnus maccoyii* Castelnau, 1872) is distributed across most of the southern temperate ocean and migrates extensively between 30°S and 50°S. Since *T. maccoyii* has been continually and heavily exploited, it is necessary to investigate the genetic diversity, population structure and demographic history of *T. maccoyii* for effective management and conservation. Thirty-seven gonad tissues of *T. maccoyii* were sampled from two locations, which were in the eastern Indian Ocean and the eastern Atlantic Ocean, by scientific observers onboard Korean *T. maccoyii* longline vessels in 2015. We compared 1240-bp sequences of combined mitochondrial DNA (mtDNA) from the cytochrome c oxidase subunit I (COI, 504-bp) and control region (CR, 736-bp) sequences. The pairwise fixation index (*F*_ST_) and maximum-likelihood tree showed that two clades (A and B) were formed regardless of locations. Clade A occurred more commonly than clade B in both localities: the occurrence ratio of clade A was 69% in the Indian Ocean, and 79% in the Atlantic Ocean, respectively. Our findings suggest that a historic differentiation event may have occurred in *T. maccoyii*, but recently the connectivity between the two oceans may be possible in *T. maccoyii* populations.

## Introduction

Southern bluefin tuna (*Thunnus maccoyii* Castelnau, 1872) is a large, long-lived, and highly migratory fish found throughout most southern temperate oceans except the more easterly regions of the southern Pacific (Polacheck 2012). This species is slow-growing and late-maturing relative to other tunas (Caton et al. 2000), and shifts among several habitats during ontogenetic growth (Patterson et al. 2018). The *T. maccoyii* stock has been exploited for more than 50 years and remains below 20% of the initial spawning stock biomass and the level estimated to produce maximum sustainable yield (CCSBT 2018).

When fisheries management is not based on the population structure, any changes may occur in the biological attributes, productivity, and genetic diversity of the exploited species (Ricker 1981). Therefore, a multidisciplinary approach is needed to define population or species boundary, and genetic studies can contribute valuable information in this regard (Pawson and Jennings 1996; Waldman 1999). Phylogeny and population genetic studies have been conducted for effective management of *Thunnus* species (Bartlett and Davidson 1991; Chow and Inoue 1993; Chow and Kishino 1995; Alvarado Bremer et al. 1997; Antoniou et al. 2017; Suda et al. 2019). Among genetic markers, the less variable portion of the cytochrome *c* oxidase subunit I (COI) and hypervariable portion of the control region (CR) have been broadly used to identify fish species, including tunas, and to clarify population structure (Ward et al. 2005; Paine et al. 2007; Kunal et al. 2013). Therefore, the present study compared the genetic diversity, population structure and demography of *T. maccoyii* between two main fishing areas of the Indian Ocean and the Atlantic Ocean.

## Materials and methods

### Sampling

**Figure 1.**
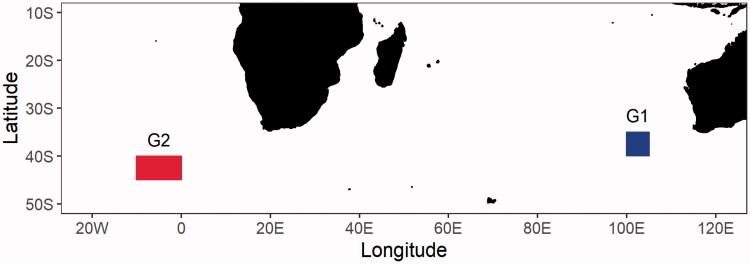
Sampling locations of *Thunnus maccoyii*, which G1 and G2 indicate samples from the eastern Indian Ocean and the eastern Atlantic Ocean, respectively.

A total of thirty-seven *T. maccoyii* gonad tissue samples were collected by scientific observers on board Korean tuna longline vessels, which were sampled in the eastern Indian Ocean (G1) and in the eastern Atlantic Ocean (G2) from April to September 2015 (Figure 1). The tissues sampled were preserved in 99% ethanol until DNA extraction. The sequences of all samples used in this study have been deposited in GenBank under accession numbers MZ222168-MZ222241.

### DNA extraction, PCR and sequencing

Genomic DNA was extracted using 10% Chelex 100 Resin (Bio-Rad, Hercules, CA, USA) from the gonadal tissues. The 736-bp fragment of the CR was amplified using the primers JEGY-F (5′-ACC GGA CGT CGG AGG TTAAA-3′) and JEGY-R (5′-TGG GCC ATA AAA TAC CCC ACTC-3′) that were newly designed to suit *T. maccoyii* in the present study. The reaction mixture for amplification of the CR had a final volume of 20 μL, including 12.3 μL of distilled water, 2 μL of 10× PCR buffer, 1.6 μL of 2.5 mM dNTPs, 1 μL of each primer, and 0.1 μL of Ex-*Taq* polymerase (Takara Shuzo, Japan). The thermal regime consisted of an initial denaturation at 94 °C for 5 min; 34 cycles of denaturation at 94 °C for 40 s, annealing at 51 °C for 45 s, extension at 72 °C for 1 min; and final denaturation at 72 °C for 5 min; the mixture was then maintained at 4 °C. The ∼650-bp fragment of COI was amplified using the primers VF2_t1 (5′-TCA ACC AAC CAC AAA GAC ATT GGCAC-3′) and FishR2_t1 (5′-ACT TCA GGG TGA CCG AAG AAT CAG AA-3′) (Ivanova et al. 2007). The reaction mixture for amplification of COI had a final volume of 20 μL, including 12.3 μL of distilled water, 2 μL of 10× PCR buffer, 1.6 μL of 2.5 mM dNTPs, 1 μL of each primer, and 0.1 μL of Ex-*Taq* polymerase (Takara Shuzo) with a Bio-Rad T100 Thermal Cycler (Bio-Rad). The thermal regime consisted of an initial denaturation at 95 °C for 5 min; 34 cycles of denaturation at 95 °C for 1 min, annealing at 52 °C for 1 min, extension at 72 °C for 1 min; and final denaturation at 72 °C for 5 min; the mixture was then maintained at 4 °C. The PCR products were purified with ExoSAP-IT (United States Biochemical Corporation USA) and then were sequenced with the ABI PRISM BigDye Terminator v3.1 Ready Reaction Cycle Sequencing Kit on an ABI 3730xl DNA Analyzer (Applied Biosystems Inc., USA).

### Data analysis

**Figure 2.**
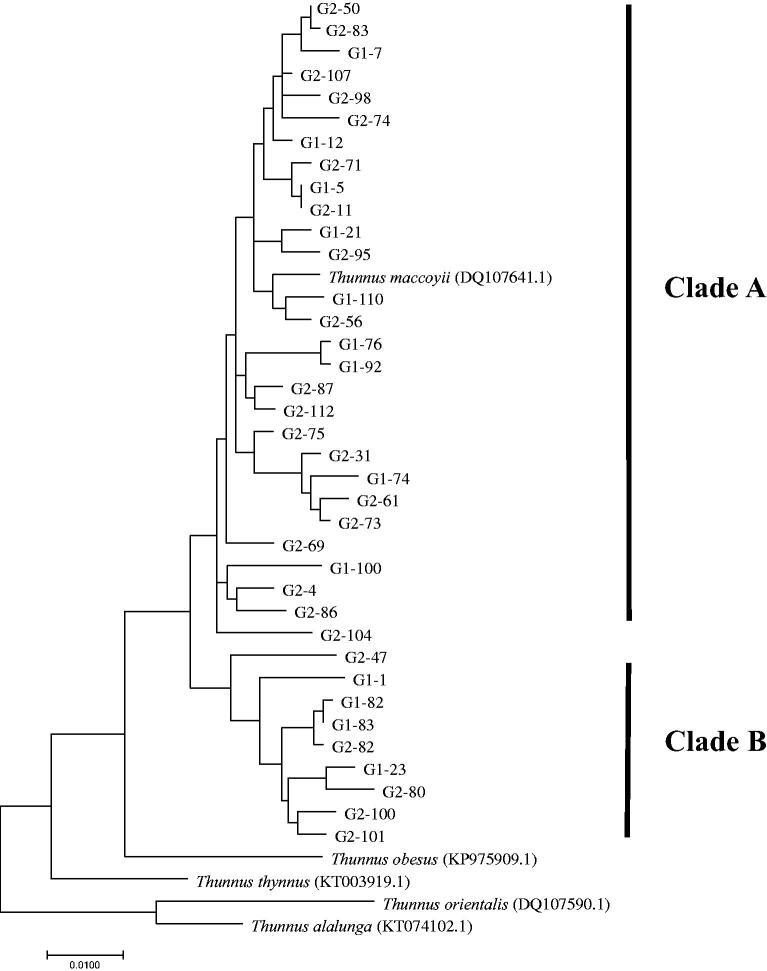
The phylogenetic tree based on the maximum-likelihood (ML) using Tamura-Nei model with 1000 bootstrap replications, for the combined mtDNA gene sequences (1240-bp) for *T. maccoyii*. G1 and G2 indicate samples from the estern Indian Ocean and the eastern Atlantic Ocean, respectively.

The generated COI and CR sequences were edited using BioEdit ver. 7.2.5 software (Hall 1999), and aligned using Clustal W program (Thompson et al. 1994) with previously determined sequences of *T. maccoyii* deposited at the National Center for Biotechnology Information (NCBI), USA (Figure 2). DnaSP ver. 5.10.01 software (Librado and Rozas 2009) was used to determine mitochondrial DNA (mtDNA) haplotypes. For each specimen, the number of haplotypes, polymorphic sites, transitions, transversions, haplotype diversity (*h*) (Nei 1987) and Nucleotide diversity (*π*) (Nei and Li 1979) were estimated to investigate the genetic diversity using Arlequin ver. 3.5.1.2 software (Excoffier et al. 2005). The phylogenetic analyses were performed by the maximum likelihood method using Tamura-Nei model (Tamura and Nei 1993) with 1000 bootstrap replications, and MEGA 7 software was used for these analyses. Pairwise fixation index (*F*_ST_) was calculated to investigate the genetic differentiation between the populations, and using Arlequin. A haplotype network was inferred from the median-joining network constructed using Network ver. 4.6.1.3 software (Fluxus Tech, USA). Evidence of population expansion was tested with Fu's *F*s (Fu 1997) and neutrality tests for equilibrium in mutational drift were carried out by Tajima's *D* (Tajima 1989) using Arlequin. Past demographic parameters were estimated, including *τ* which is time since expansion (Li 1997) and *θ*_0_ and *θ*_1_ which mean *θ* for before and after population expansion (Rogers and Harpending 1992). The value for *τ* was transformed by the equation *τ* = 2*ut* to estimate the real time since expansion (Rogers and Harpending 1992), where *u* is the mutation rate for the whole sequence and *t* is the time since expansion. The mean mutation rate of 3.6% per nucleotide per million years was used, based on Donaldson and Wilson (1999) and Mandal et al. (2012). Furthermore, Harpending's raggedness index (*Hri*) (Harpending 1994) and the sum of squared deviations (SSD) was calculated using Arlequin.

## Results and discussion

We obtained 504-bp sequences of the COI region and 736-bp sequences of CR from 37 *T. maccoyii* individuals. The COI sequences from two localities identified 37 haplotypes, and 13 polymorphic sites were detected, with 12 transitions and one transversion. The CR sequences identified 37 haplotypes, and 217 polymorphic sites were detected, with 187 transitions and 28 transversions. Haplotype diversities (*h*) were 1.000 in both localities and markers, while nucleotide diversities (*π*) differed greatly between markers: 0.002 in COI, but 0.033–0.039 in CR. The COI and CR genes showed that the *T. maccoyii* population had consistent haplotype and nucleotide diversities between two localities, indicated by the high level of haplotypes and the low level of nucleotides. And for the combined gene the *h* value was 1.000 and the *π* value ranged from 0.017 to 0.020 (Table 1). Therefore, *T. maccoyii* showed high levels of haplotype diversity in COI, CR and the combined sequences, while the nucleotide diversity was low.

**Table 1.** mtDNA sequence variability in the cytochrome c oxidase subunit I (COI), control region (CR) and the combined genes for *Thunnus maccoyii*.

Locality	*n*	*nh*	COI		CR		The combined	
			*h*	*π*	*h*	*π*	*h*	*π*
Indian Ocean	13	13	1.000 (±0.030)	0.002	1.000 (±0.030)	0.039	1.000 (±0.030)	0.020
Atlantic Ocean	24	24	1.000 (±0.012)	0.002	1.000 (±0.012)	0.033	1.000 (±0.012)	0.017

*n* indicates number of individuals; *nh*: number of haplotypes; *h*: haplotype diversity; *π*: nucleotide diversity.

The levels of haplotype and nucleotide diversities for CR sequences in the *T. maccoyii* were higher than for CR sequences in Atlantic bluefin tuna (*T. thynnus*) (0.991 and 0.015 (Carlsson et al. 2004), and 0.987 and 0.018 (Boustany et al. 2008), respectively). On the other hand, the nucleotide diversity of *T. maccoyii* was lower than that for Atlantic bonito (*Sarda sarda*) (0.051–0.071, Viñas et al. 2004b). Alvarado Bremer et al. (1997) reported a nucleotide diversity of 0.034 for *T. maccoyii* mtDNA CR, which similar to that of *T. maccoyii* in the present study. As for the combined mtDNA sequence, *T. maccoyii* showed lower nucleotide diversity compared to other *Thunnus* species (0.017–0.072, Alvarado Bremer et al. 1997), albacore tuna (*T. alalonga*) (0.054, Viñas et al. 2004a), and little tunny (*Euthynnus alletteratus*) (0.057, Alvarado Bremer and Ely 1999). High genetic diversity may be due to large population size, environmental heterogeneity, and life-history traits that allow rapid population growth (Nei 1987).

The pairwise fixation index (*F*_ST_) between the two localities did not have statistically significant values, so the indices between the two clades (A and B) were observed (Figure 2). The *F*_ST_ value between the clades was 0.308 (*p* < 0.001) based on the combined sequences (Table 2). The genetic distances of the combined sequences were 1.5–3.3% between the two clades, and 0.0–2.7% and 0.1–2.9% within clade A and B, respectively. Clades A and B appeared in both localities; the occurrence ratios of clade A were 69% in the Indian Ocean and 79% in the Atlantic Ocean, and the ratios of clade B were 31% in the Indian Ocean and 21% in the Atlantic Ocean. Although the haplotype networks showed no geographical structure for *T. maccoyii*, it was found there is the existence of two distinct clades in *T. maccoyii*, suggesting that a historic differentiation event may have occurred between clades A and B (Table 2). However, recently the connectivity between the two clades may be possible since there are two different mtDNA clades in *T. maccoyii*, but they are mixed in both the Indian and Atlantic Oceans (Figure 2). These results can be inferred that, in the past, the two clades separated from each other and formed independent clade through a unique evolutionary history, but recently the barriers between the two clades have disappeared, leading to reconnecting event. However, in order to verify the event, it is necessary to investigate whether reproductive isolation works between these clades by the microsatellite analysis.

**Table 2.** Analysis of molecular variance (AMOVA) results for the genetic structure of *T. maccoyii* based on the combined mtDNA sequence.

Source of variation	Variance	Percentage of variation	*F*_ST_	*p* Value
Between clades	5.047	30.84	0.308	<0.001
Within clades	11.317	69.16		

Tajima's *D* and Fu's *F*_s_ values for the two clades were negative, and for clade A Fu's *F*_s_ was significantly negative (*p* < 0.001) (Table 3), which means a sudden expansion in population size. Mismatch analysis showed that Fu's *F*_s_ and Tajima's *D* had a better agreement for clade A than clade B. The mean *τ* value (20.486) of two clades indicates that the sudden population expansion is estimated to have occurred 229,461 years ago.

**Table 3.** Summary of the combined mtDNA sequence variability for two clades of *T. maccoyii*.

Clade	*n*	*ti*	*tv*	*π*	Tajima's *D*	Fu's *F_S_*	*τ*	*θ*_0_	*θ*_1_	*Hri*	SSD
A	28	104	19	0.017	−1.129	−12.659*	20.973	1.846	366.338	0.0085	0.0031
B	9	67	11	0.020	−0.651	−0.985	20.000	14.400	3794.991	0.0432	0.0248
Mean	18.5	85.5	15	0.019	−0.890	−6.822	20.486	8.123	2080.665	0.0258	0.0140

*Significant at *p* < 0.001.

*n* indicates number of individuals; *ti*: number of transitions; *tv*: number of transversions; *π*: nucleotide diversity; *τ*: moment estimator, *θ*_0_ and *θ*_1_: mismatch distribution parameter estimates; *Hri*: Harpending’s raggedness index; SSD: sum of squared differences from mismatch analyses.

## Data Availability

The data that support the findings of this study are openly available in [NCBI] at [https://www.ncbi.nlm.nih.gov/], reference number [MZ222168-MZ222241].
